# Ovarian Carcinosarcoma and Response to Immunotherapy

**DOI:** 10.7759/cureus.37149

**Published:** 2023-04-05

**Authors:** Muhammad Daniyal, Anamm S Polani, Marcy Canary

**Affiliations:** 1 Internal Medicine, Bassett Hospital, Cooperstown, USA; 2 Hematology and Medical Oncology, Bassett Hospital, Cooperstown, USA

**Keywords:** carcinosarcoma, lynch syndrome, clinical case report, chemotherapy response, chemotherapy, molecular genetic testing, cancer immunotherapy, ovarian carcinosarcoma

## Abstract

Ovarian carcinosarcoma (OCS) is an uncommon and highly aggressive subtype of ovarian cancer. This form of cancer is characterized by limited treatment options and a poor prognosis. In this report, we present a case study of a 64-year-old female diagnosed with stage III OCS, who underwent debulking surgery and adjuvant chemotherapy, followed by immunotherapy, with encouraging outcomes. Despite the availability of diverse chemotherapy options, the prognosis for patients with OCS remains grim. However, the present case study of a 64-year-old female with OCS illustrates the promising outcomes achieved with immunotherapy. Additionally, this case highlights the significance of microsatellite instability testing in guiding treatment decisions for ovarian cancers of this nature.

## Introduction

Ovarian cancer is ranked as the fifth leading cause of cancer-associated deaths in women [1]. While the treatment and management of ovarian cancer mainly depend on multiple histologic types with various specific molecular and clinical characteristics, a majority of ovarian cancers constitute the epithelial subtype [2]. Of note, ovarian carcinosarcoma (OCS) accounts for 1-4% of primary ovarian tumors [3]. OCS is characterized as a complex, biphasic tumor that comprises both sarcomatous and carcinomatous elements, making it highly malignant [3]. OCS is associated with higher rates of mortality with two-thirds of patients being diagnosed at clinically advanced (III-IV) stages [4]. As such, OCS mainly presents in post-menopausal women with pelvic and/or abdominal pain, early satiety, bloating, abdominal distention, and a large tumor with hemorrhage and necrosis [5,6].

Multiple prognostic factors have been associated with poor outcomes in patients with OCS such as age, advanced stage at presentation, and suboptimal surgical resection [5]. Some other reports have also indicated the use of adjuvant chemotherapy to be more beneficial [7]. However, definitive conclusions have not been made due to the limited number of patients in these retrospective studies. Given the rarity of OCS, poor prognosis, and lack of constitutive treatments, we describe a patient treated for OCS who had an objective response to pembrolizumab, i.e., an immune checkpoint inhibitor.

## Case presentation

**Figure 1:**
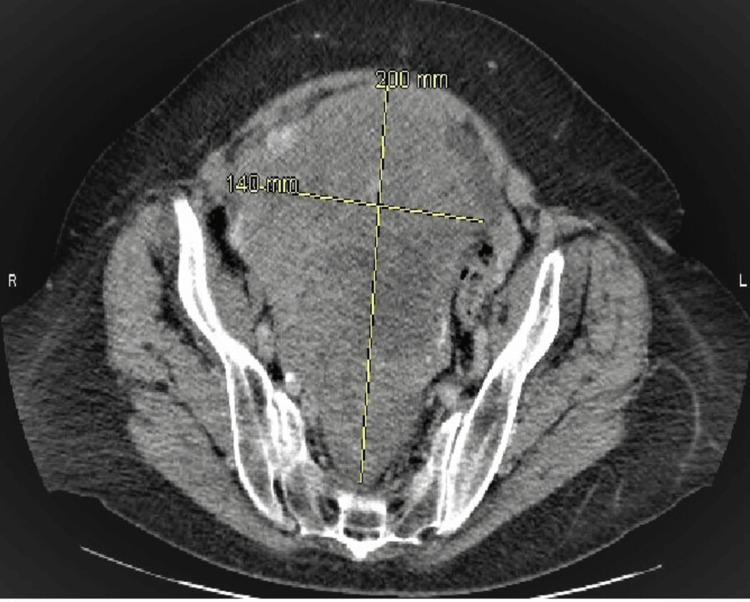
Large heterogeneously enhancing mass in the pelvis extending up to the mid-abdomen, above the umbilicus, and measuring 20 (AP) X 14 (TR) x 29 (CC) cm. AP: anteroposterior; TR: transverse; CC: craniocaudal.

A 64-year-old European descent female with a past medical history of hyperthyroidism, deep vein thrombosis on Eliquis, and microcytic iron deficiency anemia presented with loss of appetite associated with an increase in abdominal girth and pain in 2017. Family history also included a mother who died from ovarian cancer at the age of 53 years and her father who had prostate cancer. She noticed a pelvis mass enlarging in size around the same time, which was confirmed by computed tomography (CT) scan, as shown in Figure 1, followed by explorative laparotomy, total abdominal hysterectomy with bilateral salpingo-oophorectomy, small bowel resection with anastomosis, and omentectomy in 2017. She had a suboptimal debulking surgery, as she had a necrotic tumor involving her entire pelvic floor and small bowel mesentery. Her pathology report after surgery confirmed the diagnosis of stage III OCS.

**Figure 2:**
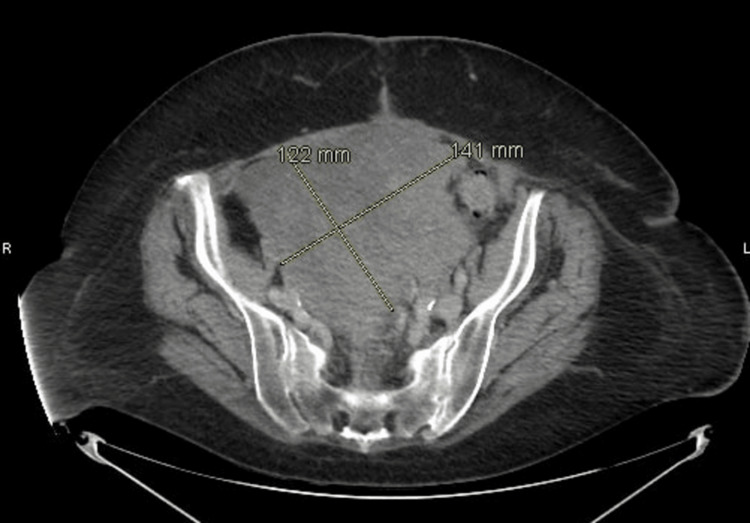
Interval enlargement of previously known large, lobulated, and heterogeneous mass in the central pelvis now measuring 16.2 x 14.1 x 12.2 cm.

The patient was started on dose-dense carboplatin and paclitaxel (carboplatinum every three weeks and 80 mg paclitaxel per meter square weekly). However, the patient was not able to tolerate the therapy, and her drug regimen was changed to less frequent dosing every three weeks, which had a fair tolerance by the patient. Her treatment response was followed up by monitoring cancer antigen (CA) 125, which started to rise toward her 6th cycle to the upper normal range, prompting a repeat CT, which showed improvement in prior residual mass, but, unfortunately, a new 11.5 cm mass was visualized. The patient was then switched to single-agent bevacizumab in 2018, which worked well for her initially, causing a decrement in pain and CA 125 levels. However, two months into treatment, her CA 125 started increasing again and a repeat CT in June 2018 showed progressive disease, as shown in Figure 2.

The patient had a significant decline in functional status and chose to undergo palliative radiation, which was completed in August 2018 followed by starting pembrolizumab 200 mg every three weeks in September 2018, after her original surgical specimen was positively tested for microsatellite instability (MSI) and was found to have a loss of nuclear expression of MutS homolog (MSH) 6. She functionally improved and remained on therapy with a stable pelvic mass, until June 2021 when she had multiple episodes of small bowel obstruction (SBO) along with elevated CA 125 levels of 12, which later self-resolved back to single digits without any change of treatment. However, the combination of recurrent SBO and rising CA 125 levels was attributed to the likely progression of the disease to possibly involve omentum, prompting the addition of cyclophosphamide to pembrolizumab, which the patient did not initiate until September 2021 due to apprehension of taking additional medicine. She presented again with SBO in November 2021 and underwent surgical exploration with two ventral hernias and no adhesions or peritoneal disease was found intraoperatively with the pelvic mass well walled off, ruling out the progression of the disease. Cyclophosphamide was discontinued at that point and the decision was made to continue pembrolizumab 400 mg every six weeks. The patient has been tolerating pembrolizumab well except for intermittent arthritic joint pain flares, especially knee joints. The patient eventually consented to genetic testing, which came back positive for MSH 6 confirming her diagnosis of Lynch syndrome.

## Discussion

Despite advanced surgical techniques and improved chemotherapy, overall survival has not improved for women with OCS. It is reported to be less than two years with no clear evidence to definitive guidelines for the systemic management of OCS [8]. Previously, the Gynecologic Oncology Group (GOG) published prospective studies of chemotherapy for women with OCS [9-11]. The response rates were approximately 20% for both ifosfamide and cisplatin, whereas doxorubicin exhibited minimal activity [9-11].

On the other hand, Rutledge and co-workers noted improved survival in women with OCS who received ifosfamide as part of their treatment in combination with other chemotherapeutic agents [12]. In contrast, patients (n = 31) who received carboplatin with paclitaxel were able to achieve a progression-free interval of 12 months while patients given a combination of cisplatin and ifosfamide did not [12]. Ifosfamide is associated with substantial toxicity and further work is needed to examine the patterns of chemotherapy use in women with OCS.

## Conclusions

Regardless of treatment and different proposed chemotherapy combinations, outcomes continue to be poor for women with OCS. However, this case presents a 64-year-old woman with OCS with promising results with immune therapy, supporting the importance of MSI testing in patients with these types of ovarian cancers.
